# Supplementary material for: Moving towards a strategy to accelerate cervical cancer elimination in a high-burden city—Lessons learned from the Amazon city of Manaus, Brazil
Source: PLoS One. 2021 Oct 18;16(10):e0258539. doi: 10.1371/journal.pone.0258539 (PMC8523067; doi:10.1371/journal.pone.0258539)
Supplement: S1 Table — Geographic and statistic Brazilian Institute (IBGE- 2019); **Data from SIB/ANS/MS (http://www.ans.gov.br/anstabnet/cgi-bin/tabnet?dados/tabnet_tx.def)—12/2020 and population—DATASUS/MS—2012; *** Data from: SINANNET/GEVEP/DEVAE (2016); # Data from 2017; & Data from PNUD/IPEA/FJP, 2010 and quote 1 American dollar = R$ 5.56. (DOCX) [file pone.0258539.s001.docx]

| Characteristics | |
| --- | --- |
| Total area (km^2^) | 11,401 (100%) |
| Urban area | 427 (3.7%) |
| Rural area | 10,974 (96.3%) |
|  |  |
| Territorial organization (health system) | 4 urban areas  1 rural area |
|  |  |
| Total population* | 2,182,763 |
|  |  |
| Female population (ages 25 to 64) | 539,813 |
| Urban area | 537,006 (4,080.00 hab/Km^2^) |
| Rural area | 2,807 (0.99 hab/Km^2^) |
|  |  |
| Health organization system ** |  |
| Public | 69.0% |
| Private | 31.0% |
|  |  |
| HIV incidence rate (general)*** | 83.2/100,000 population |
|  |  |
| Human development index*^#^* | 0.737 |
|  |  |
| Gross national product *per capita ^&^* | $ 140.00 |
| Percentage of poverty *^&^* | 12.9% |
| Percentage of extreme poverty *^&^* | 3.7% |

*Geographic and statistic Brazilian Institute (IBGE- 2019); **Data from SIB/ANS/MS (http://www.ans.gov.br/anstabnet/cgi-bin/tabnet?dados/tabnet_tx.def) - 12/2020 and population - DATASUS/MS - 2012; *** Data from: SINANNET/GEVEP/DEVAE (2016); ^#^ Data from 2017; ^&^ Data from PNUD/IPEA/FJP, 2010 and quote 1 American dollar = R$ 5.56.
