# Supplementary material for: Moving towards a strategy to accelerate cervical cancer elimination in a high-burden city—Lessons learned from the Amazon city of Manaus, Brazil
Source: PLoS One. 2021 Oct 18;16(10):e0258539. doi: 10.1371/journal.pone.0258539 (PMC8523067; doi:10.1371/journal.pone.0258539)
Supplement: S2 Table — *A woman could have contributed more than one cytology. # Coverage in some neighborhoods was over and underestimated because women may use units from nearby neighborhoods. (DOCX) [file pone.0258539.s002.docx]

| Neighborhood | Estimated female population | Cytology exams | Cytology coverage (%)^a^ | Units/  Neighborhood | Women per screening unit^b^ |
| --- | --- | --- | --- | --- | --- |
| CIDADE NOVA | 34,773 | 5,934 | 51.20 | 10 | 3,477 |
| JORGE TEIXEIRA | 29,053 | 7,047 | 72.77 | 9 | 3,228 |
| NOVO ALEIXO | 26,604 | 5,104 | 57.56 | 13 | 2,046 |
| COMPENSA | 21,515 | 3,300 | 46.01 | 10 | 2,151 |
| ALVORADA | 19,012 | 2,670 | 42.13 | 6 | 3,169 |
| SÃO JOSÉ OPERÁRIO | 18,596 | 2,797 | 45.12 | 8 | 2,324 |
| CIDADE DE DEUS | 18,539 | 2,464 | 39.87 | 9 | 2,060 |
| NOVA CIDADE | 16,667 | 730 | 13.14 | 4 | 4,167 |
| JAPIIM | 15,810 | 2,305 | 43.74 | 6 | 2,635 |
| COROADO | 14,807 | 2,759 | 55.90 | 6 | 2,468 |
| GILBERTO MESTRINHO | 14,692 | 1,567 | 32.00 | 2 | 7,346 |
| FLORES | 14,496 | 1,624 | 33.61 | 8 | 1,812 |
| PARQUE 10 DE NOVEMBRO | 12,978 | 911 | 21.06 | 5 | 2,596 |
| TANCREDO NEVES | 12,451 | 2,825 | 68.07 | 7 | 1,779 |
| PETRÓPOLIS | 12,020 | 1,430 | 35.69 | 7 | 1,717 |
| COLÔNIA TERRA NOVA | 11,909 | 2,340 | 58.94 | 7 | 1,701 |
| MONTE DAS OLIVEIRAS | 10,371 | 1,396 | 40.38 | 5 | 2,074 |
| CENTRO | 9,804 | 860 | 26.32 | 0 | - |
| REDENÇÃO | 9,724 | 1,393 | 42.98 | 4 | 2,431 |
| ZUMBI DOS PALMARES | 8,973 | 3,842 | 128.45 | 6 | 1,496 |
| TARUMÃ | 7,578 | 1,225 | 48.50 | 4 | 1,894 |
| ARMANDO MENDES | 7,469 | 1,081 | 43.42 | 3 | 2,490 |
| SANTA ETELVINA | 6,926 | 2,316 | 100.32 | 4 | 1,732 |
| SÃO JORGE | 6,480 | 1,714 | 79.35 | 5 | 1,296 |
| ALEIXO | 6,448 | 558 | 25.96 | 0 | - |
| LÍRIO DO VALE | 6,294 | 936 | 44.61 | 3 | 2,098 |
| MAUAZINHO | 6,215 | 1,452 | 70.09 | 4 | 1,554 |
| SANTO ANTÔNIO | 5,869 | 1,153 | 58.94 | 4 | 1,467 |
| DOM PEDRO | 5,263 | 325 | 18.53 | 1 | 5,263 |
| NOVA ESPERANÇA | 5,136 | 769 | 44.92 | 4 | 1,284 |
| PLANALTO | 5,093 | - | 0.00 | 0 | - |
| SÃO FRANCISCO | 5,089 | 645 | 38.03 | 4 | 1,272 |
| CACHOEIRINHA | 5,034 | 103 | 6.14 | 0 | - |
| COLÔNIA SANTO ANTÔNIO | 4,947 | 388 | 23.53 | 2 | 2,473 |
| NOSSA SENHORA DAS GRAÇAS | 4,777 | 814 | 51.12 | 4 | 1,194 |
| SANTO AGOSTINHO | 4,776 | 351 | 22.05 | 4 | 1,194 |
| EDUCANDOS | 4,654 | 863 | 55.63 | 4 | 1,163 |
| SÃO RAIMUNDO | 4,531 | 266 | 17.61 | 1 | 4,531 |
| NOVO ISRAEL | 4,433 | 1,464 | 99.07 | 2 | 2,217 |
| DA PAZ | 4,430 | 1,475 | 99.89 | 2 | 2,215 |
| CRESPO | 4,386 | 1,935 | 132.35 | 3 | 1,462 |
| RAIZ | 3,974 | 368 | 27.78 | 0 | - |
| COLÔNIA ANTÔNIO ALEIXO | 3,942 | 1,296 | 98.64 | 4 | 985 |
| CHAPADA | 3,698 | 1,727 | 140.10 | 0 | - |
| SÃO LÁZARO | 3,602 | 273 | 22.73 | 5 | 720 |
| MORRO DA LIBERDADE | 3,422 | 1,264 | 110.82 | 4 | 855 |
| TARUMÃ-AÇÚ | 3,291 | 483 | 44.03 | 1 | 3,291 |
| VILA DA PRATA | 3,285 | 645 | 58.90 | 3 | 1,095 |
| BETÂNIA | 3,178 | 695 | 65.60 | 3 | 1,059 |
| PRAÇA 14 DE JANEIRO | 3,003 | - | 0.00 | 2 | 1,501 |
| ADRIANÓPOLIS | 2,868 | - | 0.00 | 0 | - |
| GLÓRIA | 2,519 | 1,171 | 139.43 | 6 | 420 |
| PRESIDENTE VARGAS | 2,463 | 66 | 8.04 | 0 | - |
| COLÔNIA OLIVEIRA MACHADO | 2,403 | 1,217 | 151.92 | 3 | 801 |
| SÃO GERALDO | 2,200 | 214 | 29.19 | 0 | - |
| NOSSA SENHORA APARECIDA | 2,040 | 191 | 28.09 | 0 | - |
| LAGO AZUL | 1,964 | 1,568 | 239.57 | 3 | 655 |
| SANTA LUZIA | 1,942 | 470 | 72.62 | 3 | 647 |
| PONTA NEGRA | 1,664 | - | 0.00 | 0 | - |
| PURAQUEQUARA | 1,581 | 1,214 | 230.40 | 2 | 790 |
| DISTRITO INDUSTRIAL II | 1,114 | 21 | 5.65 | 1 | 1,114 |
| DISTRITO INDUSTRIAL I | 787 | - | 0.00 | 1 | 787 |
| VILA BURITI | 661 | - | 0.00 | 0 | - |

^a^ Coverage in some neighborhoods was over and underestimated because women may use units from nearby neighborhoods. ^b^A woman could have contributed more than one cytology.
