# Supplementary figures and images for: Moving towards a strategy to accelerate cervical cancer elimination in a high-burden city—Lessons learned from the Amazon city of Manaus, Brazil
Source: PLoS One. 2021 Oct 18;16(10):e0258539. doi: 10.1371/journal.pone.0258539 (PMC8523067; doi:10.1371/journal.pone.0258539)

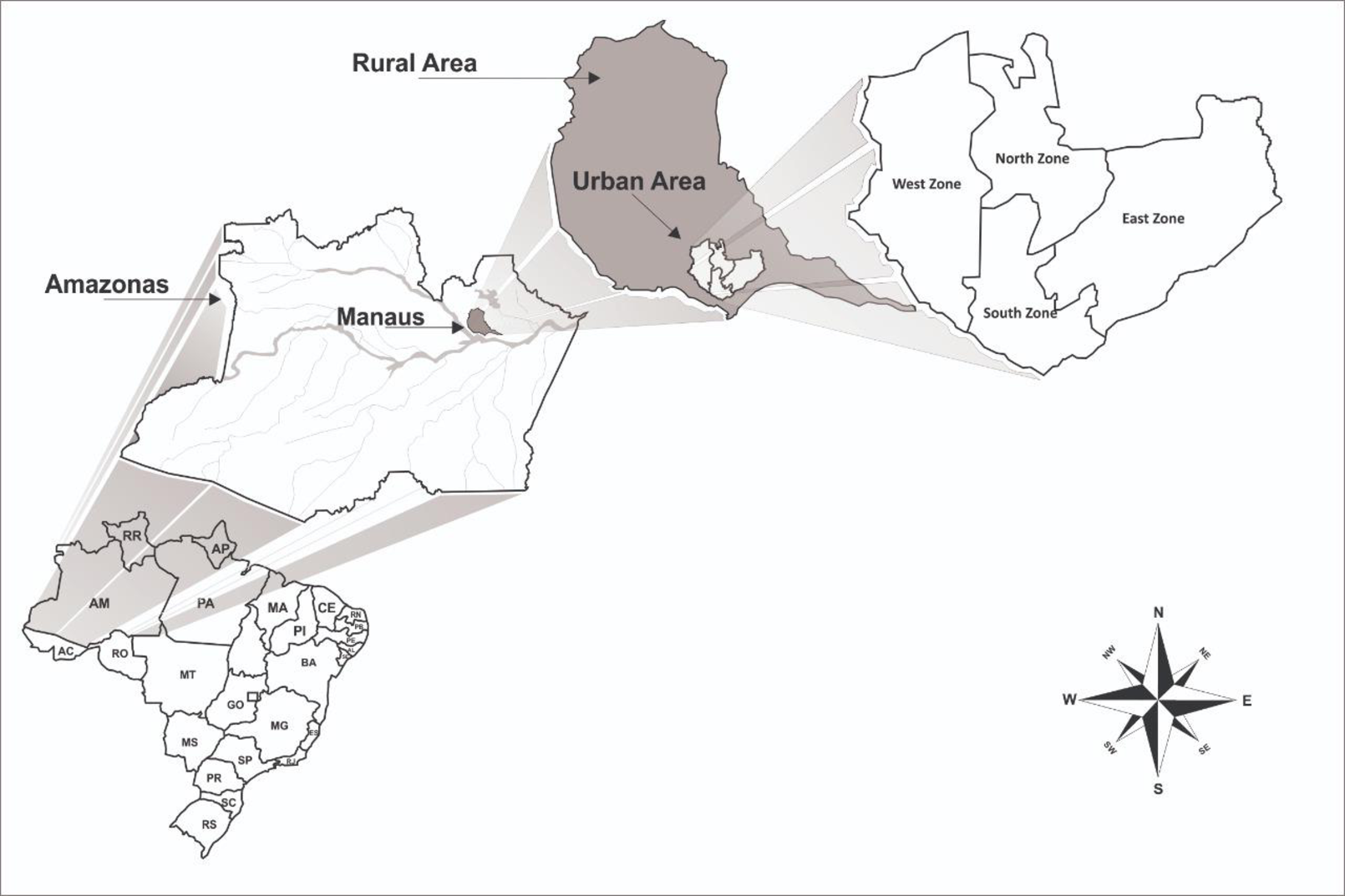

Supplement: S1 Fig — Source: adapted from IBGE cartographic base. (TIF) [file pone.0258539.s003.tif]
