# Supplementary material for: Moving towards a strategy to accelerate cervical cancer elimination in a high-burden city—Lessons learned from the Amazon city of Manaus, Brazil
Source: PLoS One. 2021 Oct 18;16(10):e0258539. doi: 10.1371/journal.pone.0258539 (PMC8523067; doi:10.1371/journal.pone.0258539)
Supplement: Attachment — Submitted filename: Dear Editor.docx [file pone.0258539.s004.docx]

Dear Editor,

This is a manuscript emphasizing the importance of vaccination, screening and post-diagnostic followup for cervical lesions. It describes the applied politics (including prevention programmes, vaccination, guideline for early lesions) and social background in Manaus (a place near Amazon, Brazil) population. The authors first defined the potential gaps (e.g: schoolbased vaccination programme covered more of the targetted population compared to clinic based approach, screening decrease cancer cases however almost %60 of the targetted population is not screened and almost half of the HSIL+lesions detected through screening is lost in followup, opportunistic screening is not enough to screen those without access to health services…). Secondly they offered some solutions reduce incidence and mortality related to cervical cancer. This is a well written manuscript, I recommend it to be published in the journal.

Regards,
