# Supplementary material for: Moving towards a strategy to accelerate cervical cancer elimination in a high-burden city—Lessons learned from the Amazon city of Manaus, Brazil
Source: PLoS One. 2021 Oct 18;16(10):e0258539. doi: 10.1371/journal.pone.0258539 (PMC8523067; doi:10.1371/journal.pone.0258539)
Supplement: Attachment — Submitted filename: Response to Reviewers.docx [file pone.0258539.s005.docx]

August 21th, 2021

Dear Dr. Emily Chenette,

We present for your consideration the response to Reviewers for our research article manuscript entitled, “**Moving towards a strategy to accelerate cervical cancer elimination in a high-burden city - lessons learned from the Amazon city of Manaus, Brazil**”.

Response to reviewers:

- We note that Figure(s) S1 in your submission contain map images which may be copyrighted. All PLOS content is published under the Creative Commons Attribution License (CC BY 4.0), which means that the manuscript, images, and Supporting Information files will be freely available online, and any third party is permitted to access, download, copy, distribute, and use these materials in any way, even commercially, with proper attribution. For these reasons, we cannot publish previously copyrighted maps or satellite images created using proprietary data, such as Google software (Google Maps, Street View, and Earth).
  - *There is no copyrighted creation. The figure was constructed for the manuscript.*
- Please clarify the sources of funding (financial or material support) for your study.
  - Financial disclosure: *We have modified our financial disclosure statement in our cover letter with this new text: ‘This research was funded in part by the United States National Cancer Institute (NIH) Intramural Research Program which participated in the study design, analysis, decision to publish and preparation of the manuscript. The authors received no specific funding for this work.’*
  - *We adjusted also Acknowledgements text to: “We thank the technical teams of the Amazonas State Health Secretary (SES-AM), Manaus Municipal Health Secretary (SEMSA/MANAUS), Oncology Control Center Foundation (FCECON), COMPREV/INCA and PNI/MOH for their assistance obtaining and analyzing data” (page 20 lines 425 to 427, (based on “Revised manuscript with track changes” file).*
- Please provide additional details regarding participant consent. In the ethics statement in the Methods and online submission information, please ensure that you have specified (1) whether consent was informed and (2) what type you obtained (for instance, written or verbal, and if verbal, how it was documented and witnessed). If your study included minors, state whether you obtained consent from parents or guardians. If the need for consent was waived by the ethics committee, please include this information.
  - *We amended the ethical statement in the Methods section of the manuscript (page 6 lines 136 to 138) (based on “Revised manuscript with track changes” file) and also added the same text to the “Ethics Statement” field in the submission form. The new text is: ‘Data were collected in collaboration with the local health authorities and the IRB waived the requirement for informed consent. For the analysis, all data were fully anonymized.’*
- Minor comments/suggestions: I would prefer to see the cytopathology labotory(ies) further depicted (page 4 line 90-91) in Material and Methods; cytopathology laboratories are also mentioned in the Discussion (page 16 line 324 and page 17 line 360) – is there one centralized laboratory or several? Likewise, the number of clinics providing colposcopy services would be interesting to know.
  - *We amended the introduction and methods sections providing information at page 4 line 72 and page 5 lines 96 to 98 (based on “Revised manuscript with track changes” file) to include more details regarding the number of colposcopy clinics and the unique centralized cytopathology laboratory, respectively.*
- Minor comments/suggestions: page 5 line 100; It would be interesting to know the number of unsatisfactory screening cytology samples as this also can describe the quality of the screening system and, also, if this varies between areas (as does the proportion of HSIL+ cytology samples).
  - *We included a new column in table 2 with the percentage of Unsatisfactory cytology samples per urban area zones (page 9 line 186) (based on “Revised manuscript with track changes” file). We also discuss the unsatisfactory cytology percentage at urban and rural zones at page page 16 lines 334 to 337 (based on “Revised manuscript with track changes” file).*
  - *We also included a new reference, number 29, regarding the Brazilian parameter for unsatisfatory cytology samples.*
- Minor comments/suggestions: Figure 2 is a bit hard to evaluate as the meaning of different colors is not provided (are different colors necessary?), some further explanation of the figure would be nice. Further discussion of the outliers, those exceeding 100% coverage, could also be of value in the discussion (as is partially for the Rural Zone already). The * on the x-axis definition is not explained anywhere.
  - *We amended Fig 2 footnote to clarify the meaning of the different colors and the asterisk. The new text, page 10 lines 203 to 207 (based on “Revised manuscript with track changes” file) “Estimated female population is represented by size of dots. Colors are used only to distinguish screening units. Dots on the x-axis indicate neighborhoods without screening units whose population attends screening units from nearby neighborhoods. *Some screening units attend to women who reside outside the area, at times resulting in coverage estimates of greater than 100% (particularly for smaller units).”*
  - *Regarding the outliers, we discuss the potential explanation in the discussion section page 16 lines 330 to 332 (based on “Revised manuscript with track changes” file).*

*We recognize the opportunity to improve some points:*

1- *The description of the methodology used to calculate vaccination coverage (page 4 lines 89 to 92);*
2- *To adjust the text of reference citation number 13 as follows:* *Instituto Brasileiro de Geografia e Estatísticas IBGE [Internet]. 2019. Available from:* [*https://cidades.ibge.gov.br/brasil/am/manaus/panorama*](https://cidades.ibge.gov.br/brasil/am/manaus/panorama) *(page 22 lines 462);*
3- *To adjust numeric formatting at S2 Table (new file attached);*
4- *To adjust text formatting for Figure 3 (new file attached);*
5- *To adjust the vaccination coverage for first and second doses for boys (10 to 13 and 14 to 15 years old) at Table 1 (page 7 line 155), at the abstract (page 2 lines 32 to 34), at the results section (page 7 lines 145 to 149), and at discussion section (page 13 lines 258 to 259) (based on “Revised manuscript with track changes” file).*

With the expectation that this manuscript will be contemplated for publication in this journal,

Best regards,

Kátia Luz Torres
